# Association between Regional Body Muscle Mass and Non-Alcoholic Fatty Liver Disease: An Observational Study Using Data from the REACTION Study

**DOI:** 10.3390/jpm13020209

**Published:** 2023-01-25

**Authors:** Jing Du, Shizhan Ma, Li Fang, Meng Zhao, Zhongshang Yuan, Yiping Cheng, Jiajun Zhao, Xiude Fan, Qingling Guo, Zhongming Wu

**Affiliations:** 1 Department of Endocrinology, Shandong Provincial Hospital, Shandong University, Jinan 250021, China; 2 Department of Endocrinology, The First Affiliated Hospital of Baotou Medical College, Baotou 014010, China; 3 Shandong Clinical Research Center of Diabetes and Metabolic Diseases, Jinan 250021, China; 4 Shandong Prevention and Control Engineering Laboratory of Endocrine and Metabolic Diseases, Jinan 250021, China; 5 Shandong Key Laboratory of Endocrinology and Lipid Metabolism, Jinan 250021, China; 6 Shandong Engineering Research Center of Stem Cell and Gene Therapy for Endocrine and Metabolic Diseases, Jinan 250021, China; 7 Department of Biostatistics, School of Public Health, Shandong University, Jinan 250021, China; 8 Department of Endocrinology, Shandong Provincial Hospital Affiliated to Shandong First Medical University, Jinan 250021, China

**Keywords:** body component, regional relative muscle mass, lower limb muscle mass, non-alcoholic fatty liver disease

## Abstract

**Background and aims:** Regional muscle distribution is associated with abdominal obesity and metabolic syndrome. However, the relationship between muscle distribution and nonalcoholic fatty liver disease (NAFLD) remains unclear. This study was to determine the relationship between regional muscle distribution and the risk and severity of NAFLD. **Methods:** This cross-sectional study ultimately included 3161 participants. NAFLD diagnosed by ultrasonography was classified into three groups (non, mild, and moderate/severe). We estimated the regional body muscle mass (lower limbs, upper limbs, extremities, and trunk) through multifrequency bioelectrical impedance analysis (BIA). The relative muscle mass was defined as the muscle mass adjusted for the body mass index (BMI). **Results:** NAFLD participants accounted for 29.9% (945) of the study’s population. Individuals with a higher lower limb, extremity, and trunk muscle mass had a lower risk of NAFLD (*p* < 0.001). Patients with moderate/severe NAFLD had a lower muscle mass of the lower limbs and trunk than patients with mild NAFLD (*p* < 0.001), while the muscle mass of the upper limbs and extremities did not differ significantly between the two groups. Moreover, similar results were found for both sexes and among different age groups. **Conclusions:** A higher muscle mass of the lower limbs, extremities, and trunk was negatively associated with the risk of NAFLD. A lower muscle mass of the limbs and trunk was inversely associated with the severity of NAFLD. This study provides a new theoretical basis for the development of individualized exercise prescriptions for the prevention of NAFLD in non-NAFLD patients.

## 1. Introduction

Nonalcoholic fatty liver disease (NAFLD) is the most prevalent form of chronic liver disorder worldwide [1], affecting over 25% of the adult population [2,3,4]. NAFLD encompasses a series of diseases ranging from steatosis to inflammatory steatohepatitis [5], cirrhosis, and hepatocellular carcinoma [6,7]. Identifying the risk factors for NAFLD is of great benefit to both prevention and therapy. Skeletal muscle, an endocrine organ, is the primary glucose disposal organ and acts as a peripheral insulin resistance (IR) regulator, which has been suggested to be associated with the pathogenesis of NAFLD. Lower muscle mass [8] and sarcopenia [9] were associated with NAFLD and liver fibrosis. Studies of children and adolescents show a similar trend [10]. Furthermore, lower muscle mass [9] and grip strength were associated with a higher risk of developing severe NAFLD [11]. There was research [12] which showed that increases in relative skeletal muscle mass over time may lead to benefits either in the development of NAFLD or the resolution of existing NAFLD.

However, previous studies have been limited by their focus on muscle mass of the total skeleton or extremities [12,13,14] and lack separate analyses of upper limb and lower limb muscle mass. Indeed, upper and lower limb muscles differ not only in quality and spatial distribution, but also in function [15] and roles in different diseases [16,17]. Studies have reported that IR occurs in lower limb muscle but not in upper limb muscle, which may be related to the dysregulation of skeletal muscle fatty acid metabolism and glucose clearance by skeletal muscle and suggested that lower limb muscle mass has great relevance in the development of type 2 diabetes [16,18]. Other studies reported that lower limbs exhibit lower muscle glucose clearance, preservation of insulin sensitivity, myosin heavy chain I fibers, and Ca^2+^ sensitivity than upper limbs [16,19,20]. These findings are important for understanding the risk factors for related disease. In contrast to previous studies, one study [21] found that the upper limb muscle mass in men had a stronger relationship with metabolic syndrome than lower limb muscle mass. This suggests that the muscle mass of different regions may be differently related to the occurrence and development of a variety of diseases. NAFLD and the diseases noted above partly share a common pathogenesis. We speculated that the association between the muscle mass of the upper or lower limbs and risks or the severity of NAFLD deserves further investigation.

Therefore, we conducted a cross-sectional study to evaluate the association between the muscle mass of various parts of the body, especially the lower limbs, and NAFLD. In addition, this study provides a new theoretical basis for the development of individualized exercise prescriptions for the prevention of NAFLD in non-NAFLD patients.

## 2. Methods and Patients

### 2.1. Participants

**Figure 1:**
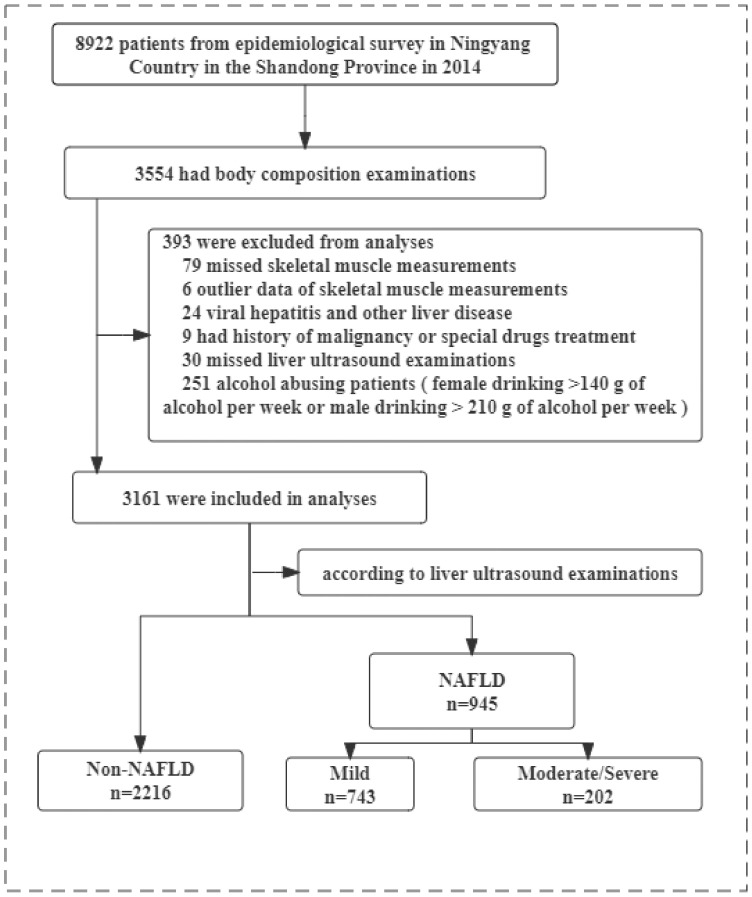
Flow diagram of the inclusion and exclusion of participants in the study. Abbreviations: NAFLD, nonalcoholic fatty liver disease.

The study population was derived from epidemiological survey data of the REACTION Study based in Ningyang County, Shandong Province, in 2014. A total of 8922 subjects aged 18 to 79 years, for whom regional muscle mass data were available, participated in the study. Trained interviewers obtained baseline data through face-to-face questionnaires, including demographic information, frequency and amount of smoking, alcohol consumption, exercise habits and amount of activity, personal and family history of disease, and substance use. The exclusion criteria were as follows: malignant diseases or chronic liver disease, including viral hepatitis, drug-related or other known chronic liver disease; alcohol consumption > 210 g/week for men or >140 g/week for women [22]; missing abdominal ultrasound data or other important information (such as muscle mass data, sex and so on); and glucocorticoid, amiodarone, or tetracycline use. After screening for the exclusion criteria, 3161 (1058 men and 2103 women) individuals were recruited for the final analysis (Figure 1).

This study conducted in Ningyang County was a branch of the REACTION study and shared a common ethics approval by the Ruijin Hospital Ethics Committee of Shanghai JiaoTong University School of Medicine (2014-52). Our study was conducted in accordance with the ethical standards proclaimed in the 1964 Declaration of Helsinki and its later amendments.

### 2.2. Data Collection

Blood pressure, height, and weight were measured according to the standards described in previous studies [23]. BMI was computed by the weight divided by the height squared (kg/m^2^). Blood samples were collected from all subjects after an overnight fast of at least 8 h. The serum biochemical parameters were measured (ARCHITECT ci16200, Abbott, IL, USA). The homeostasis model assessment for insulin resistance (HOMA-IR) was used for insulin resistance (IR) estimation and computed using the following formula: fasting glucose (mmol/L) × fasting insulin (mIU/L)/22.5 [24]. IR was defined as a HOMA-IR value above 2.5 [22].

### 2.3. Assessment of Muscle Mass and Muscle Strengh

Muscle mass and muscle strength were quantified by bioelectrical impedance analysis (BIA, InBody720, Biospace Co., Ltd., Seoul, Republic of Korea), as previously reported [23,25]. Individuals were requested to sit quietly for 10 min to achieve a normal distribution of body fluids. The muscle mass of each region (right and left upper limbs, right and left lower limbs, trunk) was measured. Muscle weakness was defined as lack of body strength, lower limb muscle strength, and upper limb muscle strength.

The muscle mass in the lower limbs was the sum of the left and right sides, as in the upper limbs. The extremity was the sum of the four limbs (kg). The muscle mass in this study is expressed as relative muscle mass. The relative muscle mass was corrected by BMI, as proposed by the Foundation for the National Institutes of Health (FNIH) Sarcopenia Project in 2014 [26]. The quartiles of relative muscle mass were calculated for each region, with Q1 and Q4 being the lowest and highest quartiles of relative muscle mass, respectively.

### 2.4. Assessment of NAFLD and Its Severity

NAFLD was diagnosed by a professional and experienced radiologist based on abdominal ultrasound (Korea GE Ultrasound LOGIQ P6) images [27].

The severity of NAFLD was divided into two groups, which were differentiated by ultrasonography: mild (diffuse increase in fine echoes in liver parenchyma) and moderate/severe (diffuse increase in fine echoes with impaired visualization or nonvisualization of the intercepted vessel borders and diaphragm) [28]. Moderate and severe fatty liver disease were merged because there was no difference in current treatment.

### 2.5. Statistical Analyses

Continuous variables with normal and skewed distributions are reported as means ± standard deviations (SDs) or medians (interquartile ranges), respectively. To test for significant differences in continuous variables, Student’s t test, the Mann–Whitney U test, and one-way ANOVA were used. All post hoc tests were corrected by the Bonferroni method. Categorical variables are expressed as quantities (percentages). The differences in categorical data were analyzed by the chi-square test. A logistic regression model was used to evaluate the relationship between muscle strength and the risk of NAFLD. Meanwhile, the relationships between regional relative muscle mass and the risk of NAFLD or the severity of NAFLD (mild, moderate/severe) were evaluated through a logistic regression model. Three logistic regression models were established and adjusted for different variables. Model 1 was adjusted for age and sex. Model 2 was further adjusted for smoking, exercise, hypertension, diabetes, and waist circumference (WC). Model 3 was further adjusted for triglycerides (TG), total cholesterol (TC), low-density lipoprotein cholesterol (LDL), high-density lipoprotein-cholesterol (HDL), alanine aminotransferase (ALT), and IR. The results are presented as odds ratios (OR) with 95% confidence intervals (CI). Further stratified analyses were performed according to sex (male and female) and age (<40, 40 to 60, ≥60). Statistical analyses were performed using IBM SPSS Statistics (Version 25.0, IBM Corp, Armonk, NY, USA), and an adjusted *p* value < 0.05 was defined as statistically significant for all analyses.

## 3. Results

### 3.1. Characteristics of the Study Population

The characteristics of the non-NAFLD and NAFLD (mild, moderate/severe) study populations are shown in Table 1. Of these participants, 945 (29.9%) had NAFLD. Individuals with NAFLD had a higher prevalence of diabetes, hypertension, and IR than those without NAFLD (all *p* < 0.001). In patients with NAFLD, the muscle mass of the lower limbs, upper limbs, extremities, and trunk were significantly lower than those in non-NAFLD individuals (all *p* < 0.001). Moreover, patients with moderate/severe NAFLD had a lower muscle mass in the lower limbs and trunk than patients with mild NAFLD (*p* < 0.05). Except for HDL, the general clinical indexes [WC, BMI, TG, TC, LDL, ALT, gamma glutamyl transpeptidase (GGT)] of the subjects were higher in the NAFLD group than in the non-NAFLD group (all *p* < 0.001).

### 3.2. Association between the Relative Muscle Mass of Each Region and the Risk of NAFLD

**Figure 2:**
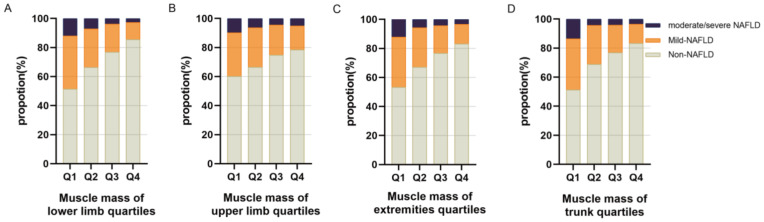
Prevalence of various degrees of NAFLD according to regional relative muscle mass. (**A**) The prevalence of non-NAFLD, mild NAFLD, and moderate/severe NAFLD according to the muscle mass of lower limb quartiles in the entire study population. (**B**) The prevalence of non-NAFLD, mild NAFLD, and moderate/severe NAFLD according to the muscle mass of upper limb quartiles in the entire study population. (**C**) The prevalence of non-NAFLD, mild NAFLD, and moderate/severe NAFLD according to the muscle mass of extremity quartiles in the entire study population. (**D**) The prevalence of non-NAFLD, mild NAFLD, and moderate/severe NAFLD according to the muscle mass of trunk quartiles in the entire study population. Abbreviations: NAFLD, nonalcoholic fatty liver disease; Q1, the lowest quartile; Q4, the highest quartile.

As shown in Figure 2, the relative muscle mass of each region was divided into four quartiles. For the lower limbs (Figure 2A), the risk of NAFLD decreased significantly with the increasing relative muscle mass (*p* < 0.001 in all quartiles). Additionally, the proportion of patients with moderate/severe NAFLD decreased from relative muscle mass Q1 to Q4. The trend was consistent across other muscle regions, including the upper limbs (Figure 2B), extremities (Figure 2C), and trunk (Figure 2D).

**Figure 3:**
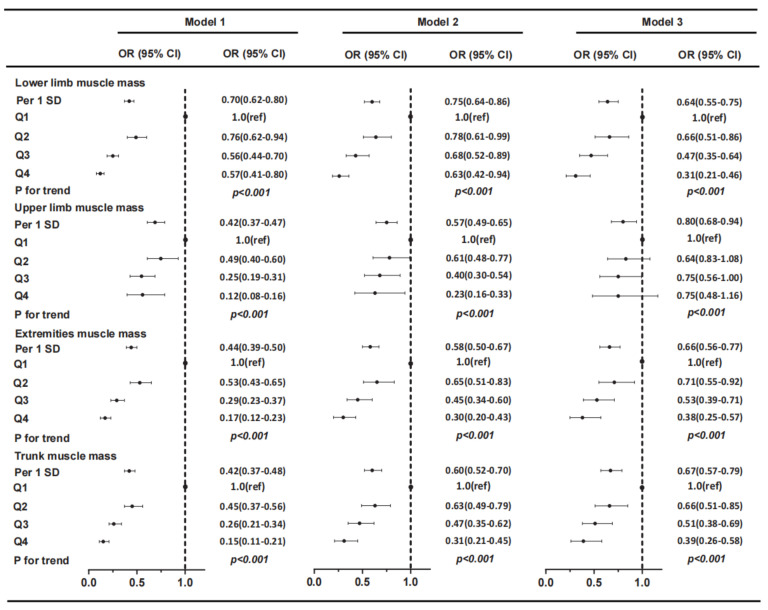
Relative muscle mass per 1 SD and per quartile and the risk of NAFLD. Model 1: adjusted for age and sex. Model 2: Model 1 + smoking, exercise, hypertension, diabetes, and WC. Model 3: Model 2 + TG, TC, LDL, HDL, ALT, and IR. Abbreviations: SD, standard deviation; NAFLD, nonalcoholic fatty liver disease; OR, odds ratio; CI, confidence interval; Q1, the lowest quartile; Q4, the highest quartile; WC, waist circumference; TG, triglycerides; TC, total cholesterol; LDL-C, low-density lipoprotein cholesterol; HDL-C, high-density lipoprotein cholesterol; ALT, alanine aminotransferase; HOMA-IR, homeostasis model assessment of insulin resistance.

**Figure 4:**
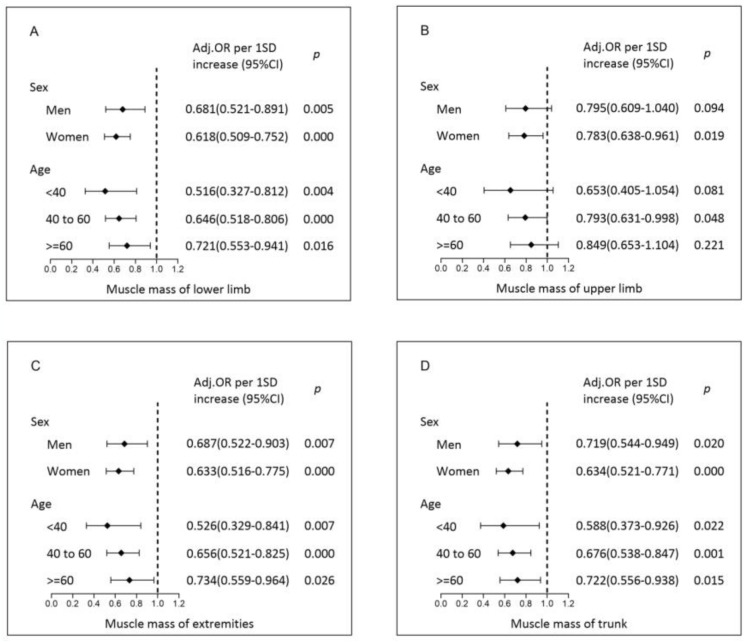
Multivariate and stratified associations between regional relative muscle mass and NAFLD by sex and age. Models were adjusted for smoking, exercise, hypertension, diabetes, and WC, TG, TC, LDL, HDL, ALT, and IR (except for sex and age, respectively). The results are presented according to muscle distribution ((**A**) muscle mass of the lower limbs; (**B**) muscle mass of the upper limbs; (**C**) muscle mass of the extremities; (**D**) muscle mass of the trunk). Abbreviations: NAFLD, nonalcoholic fatty liver disease; OR, odds ratio; CI, confidence interval; WC, waist circumference; TG, triglycerides; TC, total cholesterol; LDL-C, low-density lipoprotein cholesterol; HDL-C, high-density lipoprotein cholesterol; ALT, alanine aminotransferase; HOMA-IR, homeostasis model assessment of insulin resistance.

As shown in Figure 3, in the multivariate logistic regression model, the muscle mass of the lower limbs, upper limbs, extremities, trunk, and their respective quintiles were negatively correlated with NAFLD; the muscle mass of the lower limbs had the greatest significance, and these associations remained consistent after adjustment for age and sex (Model 1), further adjustment for smoking, exercise, hypertension, diabetes, and WC (Model 2), and further adjustment for TG, TC, LDL, HDL, ALT, and IR (Model 3). However, this trend in the upper limbs was attenuated after adjustment for confounding factors. In Model 3, the prevalence of NAFLD decreased by 36% per 1 SD incremental increase in the muscle mass of the lower limbs (OR per 1 SD increment 0.64, 95% CI 0.55–0.75, *p* < 0.001), decreased by 34% per 1 SD incremental increase in the muscle mass of the extremities (OR per 1 SD increment 0.66, 95% CI 0.56–0.77, *p* < 0.001), and decreased by 33% per 1 SD incremental increase in the muscle mass of the trunk (OR per 1 SD increment 0.67, 95% CI 0.57–0.79, *p* < 0.001). When data were stratified by age and sex (Figure 4), similar results and trends were found for the association of regional muscle mass with the risk of NAFLD in both male and female cohorts and across age groups. The muscle mass of the lower limbs, upper limbs, extremities, and trunk was significantly associated with the risk of NAFLD. The relationship between muscle strength and NAFLD disease showed the same trend as muscle mass and NAFLD disease. Corresponding analysis on the relationship between muscle strength and NAFLD disease was included in a Supplementary Table S1.

### 3.3. Association between Each Regional Relative Muscle Mass and the Severity of NAFLD

**Figure 5:**
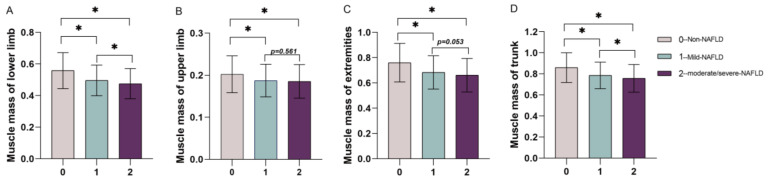
The levels of regional relative muscle mass by the degree of NAFLD. (**A**) Muscle mass of the lower limbs by the degree of NAFLD on liver ultrasound results. (**B**) Muscle mass of the upper limbs by the degree of NAFLD on liver ultrasound results. (**C**) Muscle mass of the extremities by the degree of NAFLD on liver ultrasound results. (**D**) Muscle mass of the trunk by the degree of NAFLD on liver ultrasound results; * *p* value < 0.001. Abbreviations: NAFLD, nonalcoholic fatty liver disease; 0, non-NAFLD; 1, mild-NAFLD; 2, moderate/severe NAFLD.

Of the 945 patients with NAFLD, 743 had mild NAFLD, and 202 had moderate/severe NAFLD. As shown in Figure 5, with the onset of NAFLD and the increasing severity of NAFLD (mild, moderate/severe), the levels of muscle mass of the lower limbs (Figure 5A) and trunk (Figure 5D) showed significant decreasing trends, with the association with the lower limbs being more significant. However, there were no significant differences between mild and moderate/severe NAFLD and the muscle mass of the upper limbs (Figure 5B) and extremities (Figure 5C) (*p* values were 0.561 and 0.053, respectively).

As shown in Table 2, after adjustment for sex and age in the basic model, a 1 SD increase in the muscle mass of the lower limbs, upper limbs, extremities, and trunk was associated with a 49%, 28%, 47%, and 52% lower risk of progression from mild to moderate/severe NAFLD, respectively. In the multivariate model, the risk of progression from mild to moderate/severe decreased by 40% per 1 SD increment in the muscle mass of the lower limbs (OR per 1 SD increment 0.60, 95% CI 0.43–0.84, *p* < 0.001), decreased by 39.0% per 1 SD increment in the muscle mass of the upper limbs (OR per 1 SD increment 0.61, 95% CI 0.44–0.84, *p* < 0.001), decreased by 42% per 1 SD increment in the muscle mass of the extremities (OR per 1 SD increment 0.58, 95% CI 0.42–0.81, *p* < 0.001), and decreased by 48% per 1 SD increment in the muscle mass of the trunk (OR per 1 SD increment 0.52, 95% CI 0.37–0.71, *p* < 0.001).

## 4. Discussion

Based on this large observational study, we characterized individuals with a higher muscle mass of the lower limb, extremities, and trunk as having a lower risk of NAFLD than individuals with a lower relative muscle mass. In addition, patients with moderate/severe NAFLD had a lower muscle mass of the lower limbs and trunk than patients with mild NAFLD.

Skeletal muscle, an endocrine organ, is closely related to the occurrence and development of liver diseases. Muscle mass can be classified by different regions according to the position of the body, such as the lower limbs, upper limbs, extremities, and trunk. The muscle in each region differs in quality, spatial distribution, and function [15]. Previous surveys have shown negative associations between muscle mass and the risk of NAFLD [8,9,10,11,12,29]. However, few studies have focused on the association between regional muscle mass and the risk or severity of NAFLD. To date, only the study by Wang et al. has proven the association between the muscle mass of the lower limbs and trunk and the risk of NAFLD [30], but the study was limited to older adults, and the results could not be generalized to the entire population. The trunk muscle contains the smooth muscle of internal organs [31], so the relationship between the muscle mass of the trunk and NAFLD is not precise or comprehensive. Unlike Wang et al. [30], we found that the muscle mass of the extremities was also inversely associated with NAFLD. Moreover, skeletal muscle mass was standardized by body weight, not BMI, in the Wang et al. [30] study. To date, there are three parameters for correcting relative muscle mass, including height [32], body weight [33], and BMI [34], because absolute muscle mass alone cannot rule out the effect of body size [35]. Previous studies have indicated that relative muscle mass corrected by height may underestimate the relationship between physical performance and muscle mass [36]. Moreover, BMI may be superior to weight after adjustment for body size [36]. Adjustment for BMI has been reported to be more closely associated with muscle weakness and physical dysfunction than other adjustment parameters [26,37]. Finally, the Foundation for the National Institutes of Health (FNIH) showed that only the adjustment for BMI [26] was significantly associated with muscle weakness [36]. Additionally, muscle mass corrected by BMI is the best predictor of low muscle strength [38]. Hence, in the current study, we used BMI to correct the muscle mass.

The exact mechanism underlying the association between relative muscle mass and the risk or severity of NAFLD has not been fully elucidated. Possible mechanisms include insulin resistance (IR), chronic inflammation, decreased exercise training [9], and myokines. IR, the major cause of NAFLD, is strongly related to ectopic fat accumulation in the liver [39]. The loss of skeletal muscle, the major insulin-responsive organ, can lead to a decrease in the insulin response and energy expenditure. However, the relationship between body region muscle distribution and NAFLD is not clear. Toshiaki Seko et al. [31] found that the muscle mass of the lower limbs, but not other muscle indexes, was independently related to IR, which may partly explain our results. Generally, the proportion of the muscle mass of the lower limbs (approximately 35%) was higher than that of the upper limbs (5% to 10%) [31]. Furthermore, the muscle mass of the lower limbs was more closely related to IR than that of the upper limbs. The muscle mass of the lower limbs was affected more by aging than that of the upper limbs [38]. In addition, the target population primarily performed farming work, and the results might differ from those of the general population. Chronic inflammation may also be an important link between decreased muscle mass and NAFLD [40]. Studies have demonstrated that regular physical exercise reduces the occurrence of hepatic steatosis and hepatic fibrosis [41], and reverses NAFLD [42]. Physical inactivity is associated with the severity of fatty liver disease, also supporting the view that increasing physical exercise can improve fatty liver disease from another prospective. Finally, myokines may also be an important link between decreased muscle mass and NAFLD. Skeletal muscle is an endocrine organ that releases myokines [43]. Myokines take part in the autocrine regulation of muscle metabolism and the endocrine regulation of other tissues and organs including the liver and adipose tissue [44].

Lower limb muscles have the largest muscle mass in the body and may, therefore, contribute to the decreased risk of NAFLD and its severity to some extent compared to the upper limbs or trunk. The muscle mass of the lower limbs is important because it is associated with the risk of knee osteoarthritis [45], metabolic syndrome [21], cardiovascular disease (CVD), and death [17,46].

The strength of this study was that muscle mass was analyzed according to different physiological regions, which allowed a relatively accurate and comprehensive analysis of the relationship between the muscle mass of different body regions and NAFLD, addressing the limitations of most previous studies and providing a theoretical basis for formulating reasonable exercise prescriptions. In addition, we were able to assess whether the associations were consistent across the age and sex subgroups. However, our results have prompted several topics to be addressed in future prospective studies, including the following: (1) studies on the association between regional muscle mass and NAFLD stratified by metabolic dysfunction after muscle loss begins and studies in other types of work populations; (2) studies to determine whether and how individuals with NAFLD should receive exercise training beyond lifestyle recommendations that targets lower limb muscles; and (3) studies that collect and analyze longitudinal data to confirm our findings.

There are several limitations of this study. First, it was a cross-sectional study and could not infer a causal relationship between regional relative muscle mass and NAFLD. Second, NAFLD was diagnosed by abdominal ultrasound instead of biopsy. However, ultrasonography is a simple and recommended method for the diagnosis of NAFLD. In addition, BIA is not the gold standard for assessing muscle mass, but it correlates well with dual-energy X-ray absorptiometry and has been verified in several studies on the body composition assessment [12]. Therefore, it is reasonable to use the BIA method to assess the muscle mass in a large population. Third, studies on upper and lower limb muscles do not distinguish the left and right sides, which may ignore the influence of the dominant side of human movement on muscle mass.

## 5. Conclusions

In conclusion, we demonstrated that the muscle mass of the lower limbs, extremities, and trunk was inversely associated with the risk of NAFLD. Moreover, the muscle mass of the lower limbs and trunk was inversely associated with the severity of NAFLD; however, no relationship was found between the muscle mass of the upper limbs and the risk and severity of NAFLD. From a clinical point of view, the lower limbs are the most significant body muscle distribution region associated with the risk and severity of NAFLD compared with the upper limbs. Maintaining lower limb muscle mass through exercise may, therefore, be a useful strategy for controlling NAFLD and its severity. Future studies should explore the causal association between regional body muscle mass, especially lower limb muscle mass, and the risk of NAFLD.

## Figures and Tables

**Table 1:** Descriptive characteristic of participants stratified by the severity of NAFLD.

Variables	Non-NAFLD (n = 2216)	NAFLD		*p*
		Mild (n = 743)	Moderate/Severe (n = 202)	
Age (years)	53.89 ± 12.65	53.59 ± 11.17	54.67 ± 11.19	0.534
Sex, male, n (%)	834 (37.6%)	167 (22.5%)	57 (28.2%)	0.000
WC (cm)	86.66 ± 9.70	93.92 ± 9.18 †	101.34 ± 9.25 ‡	0.000
BMI (kg/m^2^)	24.29 ± 3.14	27.44 ± 3.10 †	30.41 ± 3.71 ‡	0.000
Smoking (n, %)	367 (16.7%)	56 (7.5%)	21 (10.4%)	0.000
Enough exercise (n, %)	555 (25%)	172 (23.1%)	43 (21.3%)	0.311
Diabetes (n, %)	523 (23.6%)	277 (37.3%) †	105 (52.0%) ‡	0.000
Hypertension (n, %)	512 (23.2%)	209 (28.1%) †	85 (42.1%) ‡	0.000
HOMA-IR (>2.5) (n, %)	546 (26.5%)	350 (51.5%) †	137 (73.3%) ‡	0.000
TG (mmol/L)	1.02 (0.77, 1.47)	1.48 (1.03, 2.10)	1.81 (1.28, 2.82)	0.000
TC (mmol/L)	5.29 ± 1.06	5.39 ± 1.03	5.42 ± 1.10	0.021
LDL (mmol/L)	3.02 ± 0.86	3.13 ± 0.81	3.09 ± 0.90	0.012
HDL (mmol/L)	1.43 ± 0.34	1.28 ± 0.28	1.18 ± 0.28	0.000
ALT (IU/L)	15.0 (11, 20)	17 (13, 24)	23 (17, 32.75)	0.000
AST (IU/L)	20.0 (17, 24)	20 (17, 24)	22.2 (19, 27.8)	0.156
GGT (IU/L)	19.0 (15, 27)	23 (18, 31)	31.5 (23, 45)	0.000
Lower limb muscle mass	0.558 ± 0.114	0.496 ± 0.097 †	0.475 ± 0.096 ‡	0.000
Upper limb muscle mass	0.202 ± 0.044	0.187 ± 0.039 †	0.186 ± 0.040	0.000
Extremities muscle mass	0.761 ± 0.153	0.684 ± 0.132 †	0.661 ± 0.132	0.000
Trunk muscle mass	0.860 ± 0.141	0.786 ± 0.126 †	0.758 ± 0.132 ‡	0.000

Muscle mass was corrected by BMI; Data with normal distributions are expressed as means ± standard deviations, data with nonnormal distributions are expressed as median (interquartile range), data with categorical variables are expressed as number (percent). † *p* < 0.05 vs. non-NAFLD; ‡ *p* < 0.05 vs. mild NAFLD. Abbreviations: NAFLD, nonalcoholic fatty liver disease; WC, waist circumference; BMI, body mass index; IR, insulin resistance; TG, Triglycerides; TC, Total cholesterol; LDL-C, Low-density lipoprotein cholesterol; HDL-C, High-density lipoprotein-cholesterol; ALT, alanine aminotransferase; AST, aspartate aminotransferase; GGT, c-glutamyl transferase.

**Table 2:** Risk of NAFLD severity per 1 SD of regional relative muscle mass.

Variables (per 1 SD)	Age and Sex-Adjusted		Multivariate †	
	OR (95%CI)	*p*	OR (95%CI)	*p*
Lower limb muscle mass	0.51 (0.38–0.66)	0.000	0.60 (0.43–0.84)	0.003
Upper limb muscle mass	0.72 (0.55–0.94)	0.015	0.61 (0.44–0.84)	0.003
Extremities muscle mass	0.53 (0.40–0.70)	0.000	0.58 (0.42–0.81)	0.002
Trunk muscle mass	0.48 (0.37–0.64)	0.000	0.52 (0.37–0.71)	0.000

† Adjusted for age, sex, smoking, exercise, hypertension, diabetes, and waist circumference, TG, TC, LDL, HDL, ALT, and IR. Abbreviations: NAFLD, non-alcoholic fatty liver disease; SD, standard deviation; OR, odds ratio; CI, confidence interval; TG, Triglycerides; TC, Total cholesterol; LDL-C, Low-density lipoprotein cholesterol; HDL-C, High-density lipoprotein-cholesterol; ALT, Alanine aminotransferase; IR, insulin resistance.

## Data Availability

It is stored in the Endocrine Laboratory database of Provincial Hospital Shandong Provincial Hospital, Shandong University.

## Supplementary Materials

The following supporting information can be downloaded at: https://www.mdpi.com/article/10.3390/jpm13020209/s1, Table S1: Association of NAFLD and deficiency of muscle strength.

## Abbreviations

NAFLD	Non-alcoholic fatty liver disease
BIA	Bioelectrical impedance analysis
BMI	Body mass index
TG	Triglycerides
TC	Total cholesterol
LDL-C	Low-density lipoprotein cholesterol
HDL-C	High-density lipoprotein-cholesterol
ALT	Alanine aminotransferase
HOMA-IR	Homeostasis model assessment of insulin resistance
WC	Waist circumference
AST	Aspartate aminotransferase
GGT	Gamma glutamyl transpeptidase
