# Supplementary material for: Association between Regional Body Muscle Mass and Non-Alcoholic Fatty Liver Disease: An Observational Study Using Data from the REACTION Study
Source: J Pers Med. 2023 Jan 25;13(2):209. doi: 10.3390/jpm13020209 (PMC9959461; doi:10.3390/jpm13020209)
Supplement: Supplementary file 1 [file jpm-13-00209-s001.zip › jpm-2122763-supplementary.pdf]

Supplemental Table S1. Association of NAFLD and deficiency of muscle strength.

		**Model 1 OR(95%CI)**	**p**	**Model 2 OR(95%CI)**	**p**	**Model 3 OR(95%CI)**	**p**
Deficiency of body strength	No	1.0		1.0		1.0	
	Yes	5.098(4.115-6.317)	0.000	2.549(2.000-3.248)	0.000	2.286(1.762-2.965)	0.000
Deficiency of lower limb strength	No	1.0		1.0		1.0	
	Yes	6.757(4.976-9.175)	0.000	3.048(2.183-4.225)	0.000	2.546(1.791-3.621)	0.000
Deficiency of upper limb strength	No	1.0		1.0		1.0	
	Yes	1.917(0.974-3.773)	0.000	2.176(0.965-4.907)	0.061	1.404(0.492-4.002)	0.526

Model 1: adjusted for age and sex. Model 2: Model 1 + smoking, exercise, hypertension, diabetes and WC. Model 3: Model 2 + TG, TC, LDL, HDL, ALT and IR. Abbreviations: NAFLD, nonalcoholic fatty liver disease; OR, odds ratio; CI, confidence interval; WC, waist circumference; TG, triglycerides; TC, total cholesterol; LDL-C, low-density lipoprotein cholesterol; HDL-C, high-density lipoprotein cholesterol; ALT, alanine aminotransferase; HOMA-IR, homeostasis model assessment of insulin resistance.
